# TIP55, a splice isoform of the KAT5 acetyltransferase, is essential for developmental gene regulation and organogenesis

**DOI:** 10.1038/s41598-018-33213-4

**Published:** 2018-10-08

**Authors:** Diwash Acharya, Bernadette Nera, Zachary J. Milstone, Lauren Bourke, Yeonsoo Yoon, Jaime A. Rivera-Pérez, Chinmay M. Trivedi, Thomas G. Fazzio

**Affiliations:** 1 Department of Molecular, Cell, and Cancer Biology, University of Massachusetts Medical School, Worcester, MA 01605 USA; 2 Division of Cardiovascular Medicine, University of Massachusetts Medical School, Worcester, MA 01605 USA; 3 Department of Medicine, University of Massachusetts Medical School, Worcester, MA 01605 USA; 4 Department of Pediatrics, Division of Genes and Development, University of Massachusetts Medical School, Worcester, MA 01605 USA

## Abstract

Regulation of chromatin structure is critical for cell type-specific gene expression. Many chromatin regulatory complexes exist in several different forms, due to alternative splicing and differential incorporation of accessory subunits. However, *in vivo* studies often utilize mutations that eliminate multiple forms of complexes, preventing assessment of the specific roles of each. Here we examined the developmental roles of the TIP55 isoform of the KAT5 histone acetyltransferase. In contrast to the pre-implantation lethal phenotype of mice lacking all four *Kat5* transcripts, mice specifically deficient for *Tip55* die around embryonic day 11.5 (E11.5). Prior to developmental arrest, defects in heart and neural tube were evident in *Tip55* mutant embryos. Specification of cardiac and neural cell fates appeared normal in *Tip55* mutants. However, cell division and survival were impaired in heart and neural tube, respectively, revealing a role for TIP55 in cellular proliferation. Consistent with these findings, transcriptome profiling revealed perturbations in genes that function in multiple cell types and developmental pathways. These findings show that *Tip55* is dispensable for the pre- and early post-implantation roles of *Kat5*, but is essential during organogenesis. Our results raise the possibility that isoform-specific functions of other chromatin regulatory proteins may play important roles in development.

## Introduction

Regulation of chromatin structure is necessary to establish and maintain cell type-specific gene expression patterns during development. Generation of active or repressive chromatin architecture at gene enhancers and promoters is necessary for the binding and activities of lineage specific transcription factors^1,2^. On a broader scale, large chromosomal domains of active or repressive chromatin structure help to enforce gene expression patterns particular to each cell lineage^3^. Consequently, mutations in subunits of chromatin remodeling factors—multisubunit protein complexes that regulate chromatin architecture through a range of enzymatic activities—frequently result in developmental abnormalities^4^.

The lysine acetyltransferase KAT5 (originally named TIP60) is conserved throughout eukaryotes and activates gene expression through acetylation of histones H2A (and H2A variants), H4, and numerous transcription factors^5–9^. The KAT5 acetyltransferase activity is also necessary for the cellular response to DNA damage, in part by remodeling chromatin structure near sites of DNA damage^7,8,10–12^. Interestingly, KAT5 also has an essential non-catalytic role in regulation of chromatin accessibility and gene expression in embryonic stem cells and pre-implantation embryos^13^. KAT5 is a component of the 17-subunit TIP60-P400 complex, which remodels chromatin architecture not only through acetylation of histone tails, but also through incorporation of the H2A.Z histone variant into nucleosomes. Previously, individual knockdown of multiple subunits of TIP60-P400 resulted in loss of embryonic stem cell self-renewal, as well as a defect in differentiation^14,15^. Consistent with these phenotypes, mice with homozygous null mutations of the *Kat5* gene arrest development at E3.5, prior to implantation, and *Kat5*^−/−^ blastocysts fail to hatch from the zona pellucida when cultured *in vitro*^16^.

**Figure 1:**
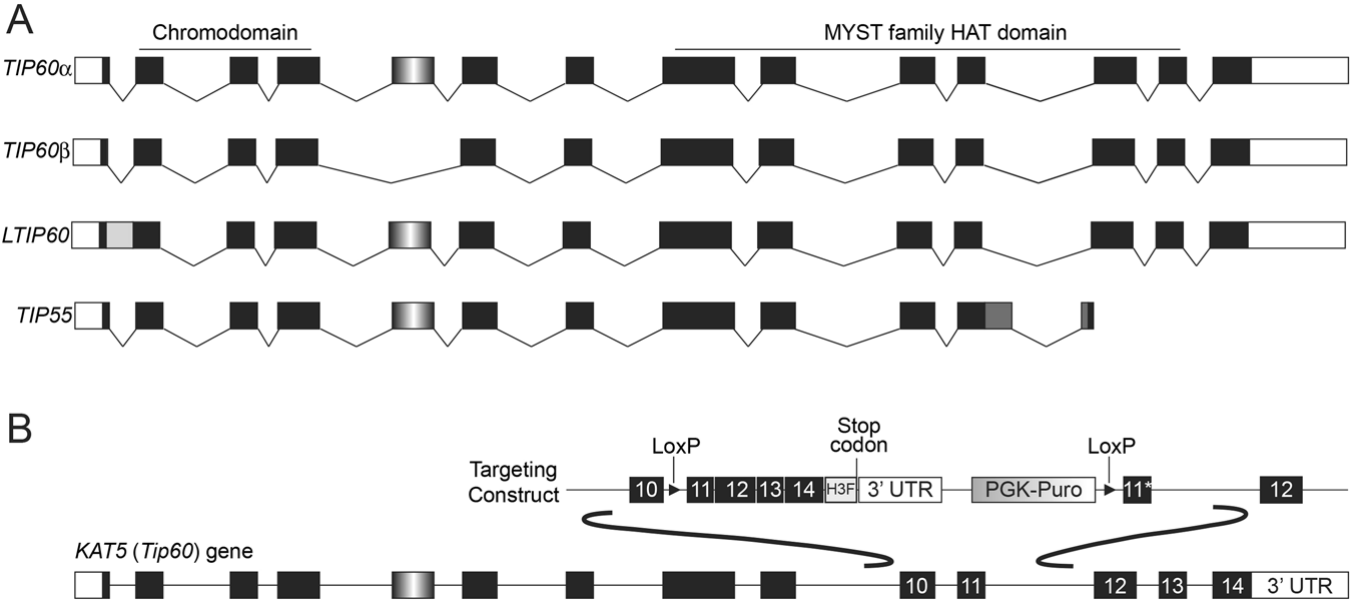
Four splice variants are expressed from the *Kat5* locus. (**A**) Variants of *Kat5* mRNA produced by alternative splicing. Black boxes: coding regions within exons; white boxes: non-coding sequences within exons; black lines: spliced introns; gray boxes: in frame intronic regions retained in specific Kat5 isoforms; gradient filled box: exon 5, which is spliced out in the Tip60β isoform. (**B**) Targeting construct used to generate both catalytic inactive *Kat5* mutant mice^13^ and *Tip55* knockout mice (described herein). The *Tip55* allele was made by removing the intronic regions retained selectively in *Tip55* and fusing the remaining exons (which are all included in all other *Kat5* isoforms).

Although most studies of *Kat5* functions have focused on a single isoform, denoted *Tip60α*, several isoforms are expressed in mouse and human by virtue of alternative splicing. Four isoforms are expressed in mouse, *Tip60α*, *Tip60β*, *LTip60*, and *Tip55*, which differ by alternative inclusion of intron 1, exon 5, or a portion of intron 11 combined with early termination of the reading frame (Fig. 1A)^17–19^. The amino acid sequences of LTIP60 and TIP55 differ from TIP60α and TIP60β near or within the critical chromodomain and MYST domain, respectively, raising the possibility that these sequence alterations affect their functions. The *Kat5* gene is broadly expressed throughout development, although it is unclear which isoforms are expressed in each tissue, owing to a lack of reagents for distinguishing the different isoproteins. Therefore, the extent to which each *Kat5* isoform functions specifically or redundantly with other isoforms is currently unknown.

Here we test the hypothesis that *Tip55* acts non-redundantly in development by specifically mutating this isoform within the mouse *Kat5* gene. We show that *Tip55* is necessary for embryonic development, with *Tip55* homozygous mutant animals dying at or around E11.5. Prior to the appearance of overt developmental phenotypes, we observe defects in both heart and neural tube, which manifest as defects in cellular proliferation and increased cell death. Transcriptomic analyses of embryos prior to the appearance of developmental defects reveal alterations in developmental pathways that contribute to numerous tissues and organ systems. These data reveal a critical function of *Tip55* in organogenesis that is distinct from the early, pre-implantation defect observed when all four *Kat5* isoforms are lost and raise the possibility that additional isoforms of *Kat5* have unique, non-redundant functions during development.

## Results

### *Tip55* is essential for embryonic development

The TIP55 protein lacks the C-terminal 124 amino acids of the other three KAT5 isoforms, TIP60α, TIP60β, and LTip60. In its place, TIP55 has a unique 103 amino acid C-terminus encoded by a portion of intron 11^17^ (Fig. 1A). This unique C-terminal region lacks amino acid sequence features of any well-established protein domain. We previously generated a multifunctional *Kat5* mutant mouse that specifically eliminates the *Tip55* isoform and can be converted to a catalytically inactive *Kat5* allele upon Cre-mediated recombination. To generate the *Tip55* null mutation, we deleted introns 11, 12, and 13, fusing exons 11–14, and in the process removing the region encoding the unique 103 amino acids of TIP55 (Fig. 1B). This allele eliminates *Tip55* expression, but does not alter the coding sequence or expression of the remaining isoforms (Fig. 1A, Fig. S1A–C), which are able to form normal TIP60-P400 complexes^13^. We previously examined the role of the *Kat5* lysine acetyltransferase (KAT) activity after Cre-mediated recombination of the *Tip55* KO cassette, and found that these KAT-deficient mutant mice progress beyond pre-implantation stages but exhibit defects at or before gastrulation^13^. However, the question of whether *Tip55* is required at any stage of development has not been addressed.

**Figure 2:**
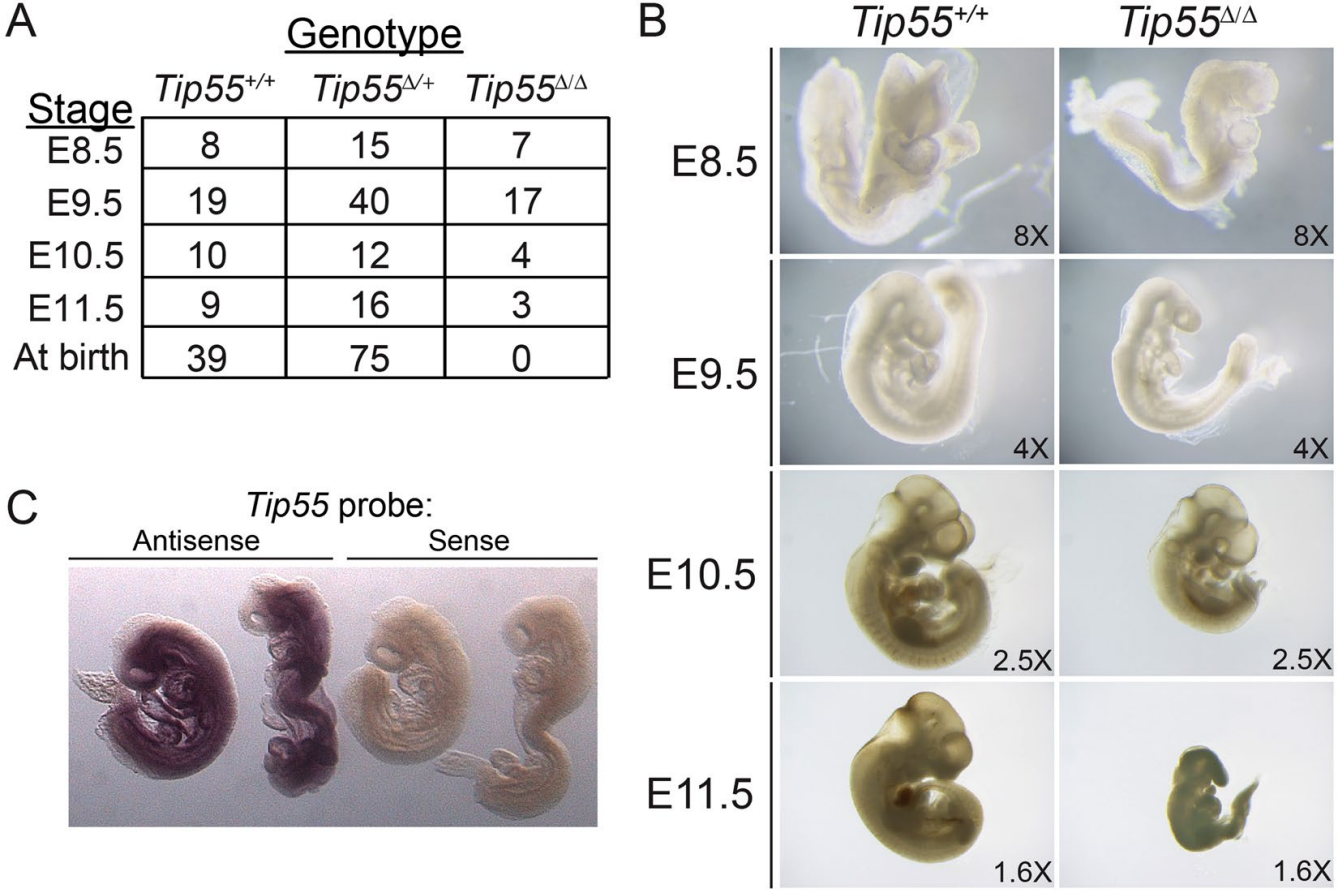
Tip55 homozygous knockout mice die at or before embryonic day 11.5. **(A)** Genotypes of embryos recovered at indicated stages of development or at birth. **(B)** Representative images of wild type (*Tip55*^+/+^) and knockout (*Tip55*^*Δ/Δ*^) embryos at stages indicated. Different magnifications as indicated (lower right) were required to image embryos at each stage, owing to differences in size. **(C)** Whole-mount *in situ* hybridization of *Tip55* transcript. Shown are wild type E8.5-E9.0 mouse embryos hybridized with antisense or sense (as a negative control) *in situ* hybridization probes corresponding to the region of intron 11 retained in the *Tip55* transcript but not found in all other *Kat5* isoforms.

To test this possibility, we intercrossed mice heterozygous for the *Tip55* mutant allele (hereafter, *Tip55*^*Δ/*+^) to generate *Tip55*^*Δ/Δ*^ homozygotes. We recovered no *Tip55*^*Δ/Δ*^ pups at birth (χ^2^ = 38.05; *P* < 0.0001), suggesting that the *Tip55* isoform is essential for embryonic development (Fig. 2A). To determine the stage at which embryonic development of *Tip55*^*Δ/Δ*^ mice was blocked, we dissected and genotyped embryos from E8.5 to E11.5. No overt phenotype was apparent at E8.5 (Fig. 2B). In contrast, *Tip55*^*Δ/Δ*^ embryos were smaller than *Tip55*^+/+^ or *Tip55*^*Δ/*+^ at E9.5, although they appeared morphologically normal (Fig. 2B). Although *Tip55*^*Δ/Δ*^ embryos could readily be recovered as late as E10.5 and occasionally as late as E11.5 (Fig. 2A), all E11.5 *Tip55*^*Δ/Δ*^ embryos lacked beating hearts, suggesting a potential cause of death. The lack of *Tip55* expression in *Tip55*^*Δ/Δ*^ homozygotes was confirmed by RT-PCR (Fig. S1). These data reveal that, unlike null mutants lacking all *Kat5* isoforms or *Kat5* KAT-deficient mutants, *Tip55*^*Δ/Δ*^ mice progress normally through early pre- and post-implantation developmental stages. However, *Tip55*^*Δ/Δ*^ mice die during mid-gestation, potentially due to a fully penetrant defect in heart development.

### *Tip55* is required for development of the heart and neural tube

Key early steps in organogenesis, including formation of the heart tube and closure of the neural tube, occur at approximately embryonic day 8 of mouse development, shortly before developmental defects are apparent in *Tip55*^*Δ/Δ*^ embryos. To investigate the possibility that *Tip55*^*Δ/Δ*^ embryos are defective in some aspects of organ formation, we first tested whether *Tip55* was expressed in normal embryos at the initial stages of organogenesis. We performed RT-qPCR for *Tip55*, as well as for two additional *Tip60* isoforms for which specific primers could be designed, *LTip60* and *Tip60*β, using RNA isolated from E7.5-E11.5 embryos. We observed *Tip55* expression at all stages, albeit to lower levels than observed for the other isoforms (Fig. S2). Furthermore, using whole-mount *in situ* hybridization and an intron 11 probe specific for the *Tip55* isoform (dark gray boxes in Fig. 1A), we observed that expression of *Tip55* was widespread in E8.5-E9.0 embryos (Fig. 2C).

**Figure 3:**
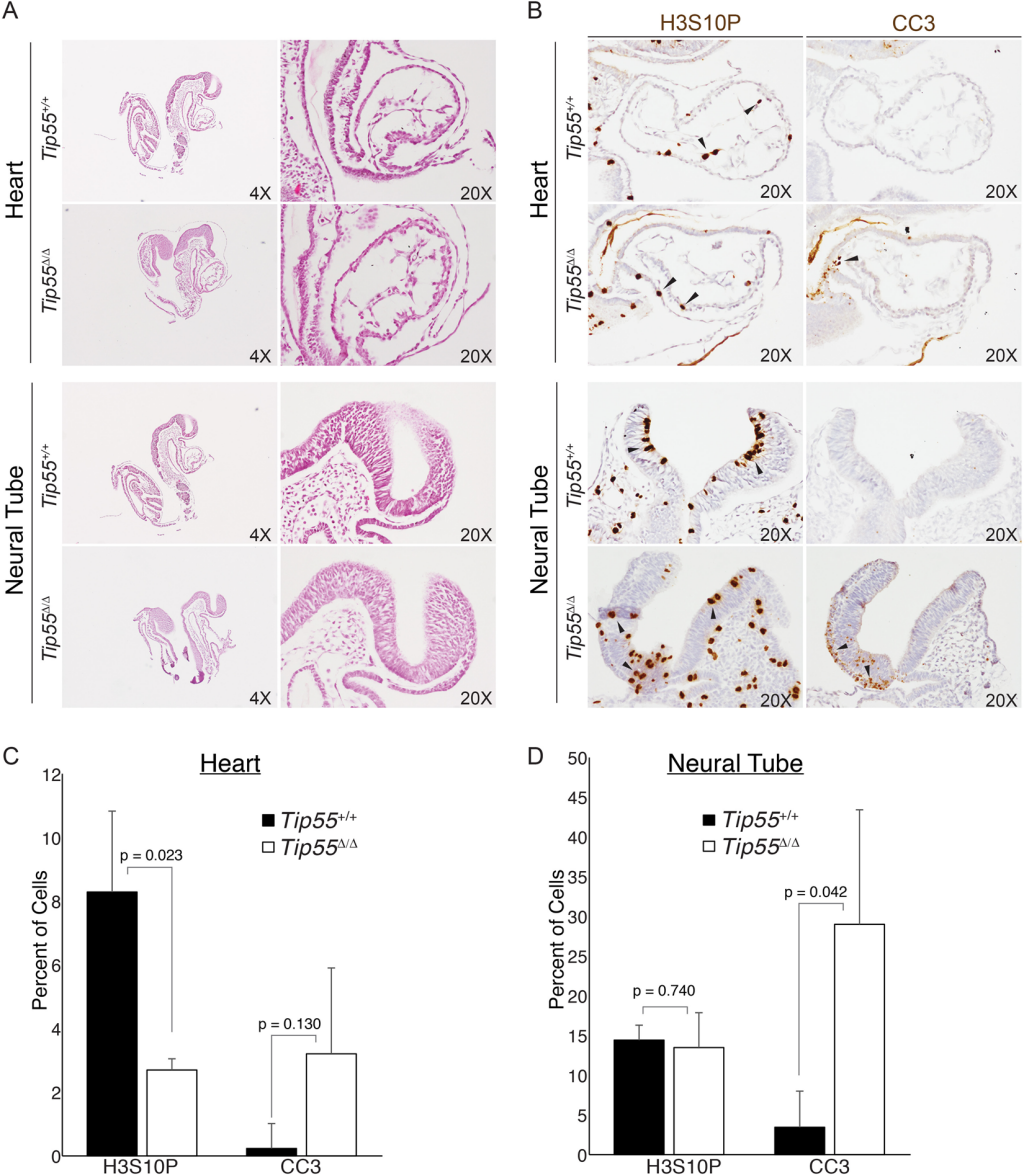
*Tip55* loss leads to defects in heart and neural tube. **(A)** Hematoxylin and Eosin (H&E) stained E8.5 embryo sections of *Tip55*^+/+^ and *Tip55*^*Δ/Δ*^ embryos, shown at multiple magnifications to reveal overall morphology (4X) or focus on heart (20X, above) and neural tube (20X, below). **(B)** Immunohistochemistry staining of cleaved caspase 3 (CC3) or Histone H3 phosphorylated on serine 10 (H3S10P) on E8.5 sections of *Tip55*^+/+^ and *Tip55*^*Δ/Δ*^ embryos. Examples of positively staining cells denoted with arrowheads. **(C**,**D)** Quantification of cells staining positively for each H3S10P and CC3 in heart (**C**) and neural tube (**D**). N = 3 embryo sections were used. P-values were calculated using a two-sided t-test.

Next, we examined several features of *Tip55*^+/+^ and *Tip55*^*Δ/Δ*^ embryos at E8.5 (prior to the appearance of developmental defects in *Tip55*^*Δ/Δ*^ mutants) in embryo sections. We observed no obvious differences in organization of the body plan in sagittal sections of multiple embryos of each genotype (Fig. 3A). We performed immunohistochemistry with antibodies against Histone H3 phosphorylated at serine 10 (H3S10P) or cleaved caspase 3 (CC3) to test for proliferating or apoptotic cells, respectively. Although most regions of *Tip55*^*Δ/Δ*^ embryos were normal, the proportion of H3S10P positive cells was significantly reduced in heart, whereas cleaved caspase 3 staining was significantly elevated in neural tube (Fig. 3B–D). (Cleaved caspase 3 staining also appeared to be higher in heart in *Tip55*^*Δ/Δ*^ embryos, but the differences were not statistically significant.) These data suggest that loss of *Tip55* results in defects in proliferation and/or increased apoptosis in multiple organs and further suggest that embryonic lethality caused by this mutation is due to defects in organ development.

We next examined whether the observed phenotypes were associated with a failure to specify the heart and neural lineages. To test this possibility, we performed immunofluorescence staining of wild type and mutant embryo sections for markers of neural lineage (SOX2) and cardiac muscle (cardiac troponin T, cTNT). As above, we stained sections from E8.5 embryos to minimize indirect effects of developmental arrest. However, we observed no difference in staining of either marker in *Tip55* mutant embryos relative to wild type (Fig. S3). These data suggest that *Tip55* loss impairs proliferation and viability of cardiac and neural progenitor cells downstream of cell type specification in both organs.

### *Tip55* is required for proliferation of MEFs *ex vivo*

**Figure 4:**
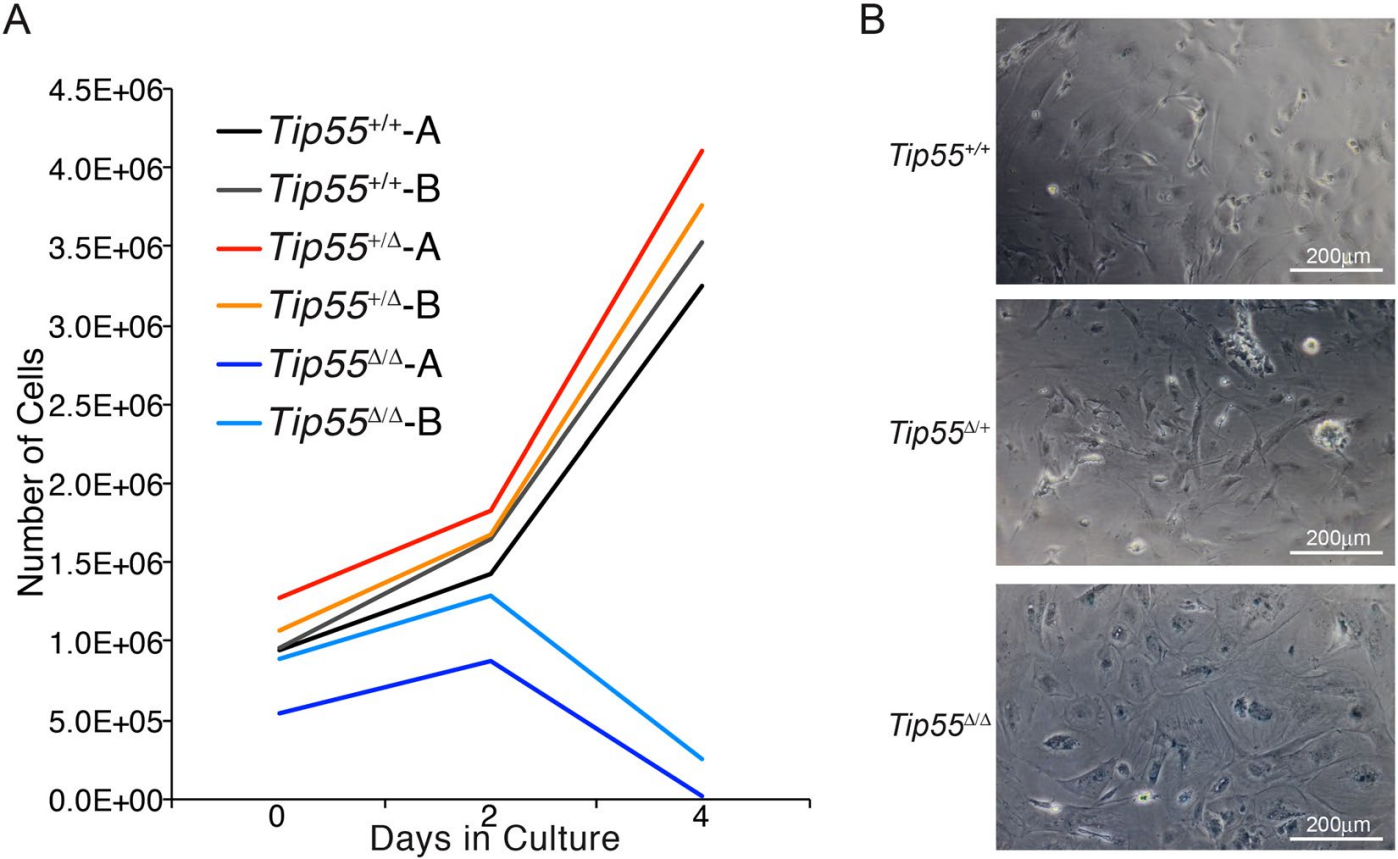
Tip55 mutant MEFs exhibit premature proliferation arrest. (**A**) Growth curve indicating numbers of cells of each genotype after growth in culture for indicated lengths of time. Cells were seeded at approximately equivalent densities. (**B**) Bright field images of mouse embryonic fibroblasts (MEFs) isolated from mouse embryos with the indicated genotypes and stained for β-galactosidase activity (blue) after five days of culture. Scale bars are indicated.

The reduced H3S10P staining of *Tip55*^*Δ/Δ*^ embryos in E8.5 heart suggested that TIP55 may promote cellular proliferation. However, a reduction in the number of proliferating cells could also result indirectly from increased cell death or other defects. Therefore, to directly test whether TIP55 was necessary for proliferation, we measured the proliferation rate and morphology of cells isolated from embryos with and without *Tip55* mutations. Fibroblasts (MEFs) were isolated from *Tip55*^+/+^, *Tip55*^+*/Δ*^*,* and *Tip55*^*Δ/Δ*^ embryos and cultured during a four-day time course. We found that *Tip55*^*Δ/Δ*^ MEFs proliferated minimally after isolation at E9.5, followed by growth arrest shortly thereafter (Fig. 4A). Furthermore, *Tip55*^*Δ/Δ*^ MEFs exhibited flattened and elongated cell morphology reminiscent of cells undergoing senescence, in contrast to *Tip55*^+/+^ and *Tip55*^*Δ/*+^ cells (Fig. 4B). Indeed, *Tip55*^*Δ/Δ*^ MEFs exhibited modest but reproducibly elevated staining for senescence-associated β-galactosidase activity compared to *Tip55*^+/+^ and *Tip55*^*Δ/*+^ MEFs (Fig. 4B). Together, these data suggest that *Tip55* is required for the proliferation of some embryonic cell types and suppression of cellular senescence.

### Drivers of organ and tissue development are misregulated in *Tip55*^*Δ/Δ*^ mice

**Figure 5:**
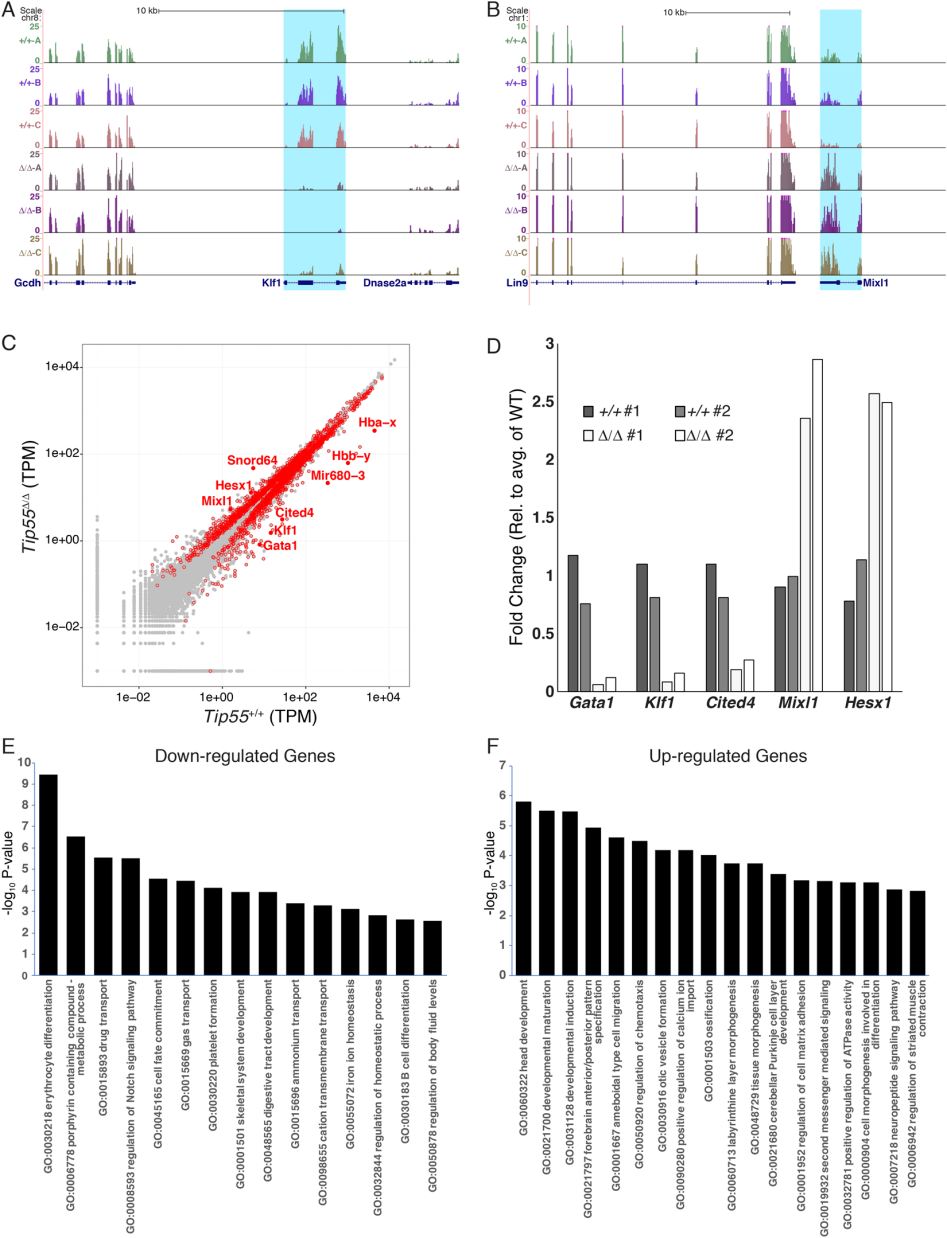
Genes necessary for organogenesis are misregulated in *Tip55* mutant embryos. **(A,B**) Browser tracks of developmental regulators (highlighted in blue) downregulated (**A**) or upregulated (**B**) in *Tip55*^*Δ/Δ*^ embryos (Δ/Δ) relative to *Tip55*^+/+^ embryos (+/+). Three biological replicate RNA-seq datasets (normalized for read number) for each geneotype were performed. **(C)** Average transcripts per million (TPM) for each genotype are shown on a log-log scale with genes significantly differentially expressed in *Tip55*^*Δ/Δ*^ embryos (posterior probability of differential expression; PPDE > 0.95) highlighted with red circles. Several genes of interest labeled with solid red dots with gene names shown. **(D)** RT-qPCR validation of indicated genes from *Tip55*^+/+^ (+/+) or *Tip55*^*Δ/Δ*^ (Δ/Δ) E8.5 embryos. Genes were selected based on differential expression (up or downregulation) in RNA-seq experiments. Expression levels in biological duplicate *Tip55*^+/+^ or *Tip55*^*Δ/Δ*^ embryos are plotted individually, relative to the average of the *Tip55*^+/+^ (which is set to 1). **(E**,**F)** Significantly enriched gene ontology (GO) categories for genes down-regulated or up-regulated significantly (PPDE > 0.95) and |log2 (Fold Change)| >0.6 are depicted in (**A**) and (**B**), respectively.

KAT5 isoforms act in part by acetylation of the N-terminal tails of histones H2A and H4 near gene regulatory regions, which promotes transcription^10^. To test whether genes required for development, cellular proliferation, or other processes were misregulated upon loss of the *Tip55* isoform, we isolated RNA from *Tip55*^+/+^ or *Tip55*^*Δ/Δ*^ embryos at E8.5, prior to the appearance of morphological defects in *Tip55*^*Δ/Δ*^ embryos (Fig. 2B), and performed RNA-seq. We observed strong concordance among three biological replicates for *Tip55*^+/+^ and *Tip55*^*Δ/Δ*^ (Fig. 5A,B). Next, we used EBseq analysis^20^ to identify genes that are significantly differentially expressed in *Tip55*^*Δ/Δ*^ embryos. We identified 2507 genes that were significantly misregulated in *Tip55*^*Δ/Δ*^ embryos (posterior probability of differential expression, PPDE > 0.95), with 278 of those genes misregulated more than two-fold (Fig. 5C and Table S1). We validated several differentially expressed genes by RT-qPCR and observed results consistent with the RNA-seq data (Fig. 5D).

To uncover classes of genes significantly misregulated in *Tip55*^*Δ/Δ*^ embryos, we identified Gene Ontology (GO) terms significantly enriched among the differentially expressed genes. These analyses demonstrated that the differentially expressed genes were highly enriched for developmental regulators and genes required for development of multiple tissue types, including blood, muscle, and brain (Fig. 5E,F). *Kat5* is necessary for proper silencing of a number of developmental regulators in embryonic stem cells^13^. Therefore, genes upregulated in *Tip55*^*Δ/Δ*^ embryos may include some that are repressed by TIP55. In addition, developmental delays in *Tip55*^*Δ/Δ*^ embryos may cause expression of genes that peak early in wild type embryos (and normally exhibit reduced expression by E8.5) to be more highly expressed in *Tip55*^*Δ/Δ*^ mutants at E8.5 as a result of their delayed or extended window of expression.

Among numerous developmentally regulated genes that were downregulated in *Tip55*^*Δ/Δ*^ embryos were factors that promote blood cell development – including key erythroid factors *Gata1* and *Klf1* – suggesting *Tip55* loss leads to a failure to initiate erythropoiesis. However, these data do not distinguish whether the effect of *Tip55*^*Δ/Δ*^ on erythroid development is due to a direct role for TIP55 at the regulatory regions of key erythroid regulators or an indirect effect on gene expression due to other developmental abnormalities (such as in heart). In sum, we conclude that *Tip55* is necessary in multiple cell and tissue types for normal expression of differentiation genes that are critical for organ development.

## Discussion

Here we have shown an essential, non-redundant role of the *Tip55* isoform of *Kat5* during post-implantation embryonic development. These findings contrast with the phenotype of a mouse mutant that lacks all four *Kat5* transcripts, which causes pre-implantation lethality at the blastocyst stage^16^, as well as a KAT-deficient mutant that exhibits defects in gastrulation^13^. The defects of *Tip55*^*Δ/Δ*^ embryos in formation of heart and neural tube suggest this mutation impairs development of multiple cell lineages, a finding that is verified by our transcriptomic data indicating mis-expression of developmental regulators of multiple tissue types. On a cellular level, *Tip55*^*Δ/Δ*^ results in a combination of reduced proliferation and increased apoptosis in several different tissues and cell types. Therefore, widespread developmental defects may result from poor proliferation or survival of cells that comprise multiple tissues. Alternatively, these cellular phenotypes may result from more complex developmental impairments owing to defects in the gene regulatory networks specific to each lineage.

The finding that *Tip55*, a poorly-studied splice variant of *Kat5*, plays specific roles in development raises the possibility that each of the other *Kat5* isoforms play non-redundant roles in development that have yet to be discovered. This raises a larger question regarding regulation of developmental gene expression in mammals – do additional chromatin regulatory enzymes that are expressed in multiple isoforms have distinct functions that are specific for each isoform? Although the functions of splice variants of a few mammalian chromatin regulatory enzymes have been addressed *in vitro*^21,22^, the generation of isoform specific mutant animals will be necessary to assess their potential functions *in vivo*. Such studies have been performed in *Drosophila* for the SWI/SNF family ATPase, *domino*, in which two splice isoforms were found to function in distinct cell types^23^. Although multiple isoforms of one of two mammalian homologs of *domino*, *Ep400*, have been identified, it remains to be tested whether they exhibit similar cell type specificity. Therefore, an effort to make and characterize isoform-specific alleles of numerous mammalian chromatin remodeling enzymes will likely provide important insights into developmental gene regulation.

## Methods

### Antibodies

Antibodies used in this study were as follows: H3S10P (9701, Cell Signaling Technologies), and Cleaved caspase 3 (9661, Cell Signaling Technologies).

### Generation of *Tip55* knockout mice

All animal experiments were approved by the Institutional Animal Care and Use Committee of University of Massachusetts Medical School (approval number A-2165). All animal procedures were performed in accordance with UMMS and NIH guidelines on animal care. The mutant mouse line with the *Tip55* deletion is equivalent to the parental line in which the *Tip60*^*ci*^ allele was generated by Cre mediated recombination of fused exons 11–14, placing a catalytically inactive version of exon 11 into the *Kat5* gene. This parental allele (prior to Cre mediated recombination) specifically lacks the *Tip55* isoform^13,14^. Mice were genotyped by PCR with primers listed in Table S2. *Tip55*^*Δ/*+^ mice were maintained as heterozygotes on an inbred FVB/N background and intercrossed to generate *Tip55*^+/+^, *Tip55*^*Δ/*+^ and *Tip55*^*Δ/Δ*^ embryos.

### RNA ***in situ*** hybridization

Whole mount *in situ* hybridization of wild type embryos were performed as previously described^24^. A unique region of Tip55 cDNA (293 bases) was used to generate Tip55 sense and anti-sense probes.

### Derivation of MEFs

MEFs were generated from E9.5 embryos as previously described^25^. Briefly, E9.5 embryos were dissected, and trypsin (0.05%) digested for 12 minutes at 37 °C. Embryos were pipetted to obtain single cells and cultured in a 12 well plate in DMEM with 10% FBS.

### Cell proliferation assays

MEFs from two independent embryos from each genotype (*Tip55*^+/+^, *Tip55*^*Δ/*+^ and *Tip55*^*Δ/Δ*^) were seeded at approximately 50% confluency and cultured in wells of a 12-well plate. The total number of cells were counted and re-plated into new 12-well plates every 48 hours for a total of four days to measure proliferation rate.

### β-galactosidase staining

MEFs were isolated from E9.5 embryos and cultured in a 12-well plate. Three days after plating, cells were sub-cultured for an additional two days followed by staining for β-galactosidase activity using a kit (Millipore KAA002) according to manufacturer’s protocol.

### Hematoxylin and Eosin (H&E) Staining

E8.5 embryos were collected and sectioned at 8μm thickness for morphological analysis as previously described^26^. Hematoxylin- and eosin- staining was performed by de-paraffinizing sections in xylene, rehydrating slides through an ethanol gradient, staining for 30 s with 30% Harris modified hematoxylin and a 30 s counterstain with eosin Y. Slides were rinsed and dehydrated with ethanol, cleared with xylene, and mounted using Vectashield mounting media.

### Immunohistochemistry and Immunofluorescence

Sections from E8.5 embryos were examined for proliferation and apoptosis defects by immunohistochemistry, following protocols described previously^26^. Briefly, sections were rehydrated through an ethanol gradient, followed by heat antigen retrieval (Buffer A, Electron Microscopy Sciences). Immunostaining was conducted using the Vectastain Elite ABC and DAB Peroxidase Substrate kit according to manufacturer guidelines. Sections were incubated with H3S10P (1:100; Cell signaling, CSG 9706) or cleaved caspase 3 (1:100; Cell signaling, CSG 9661) biotinylated primary antibodies overnight at 4 °C^26^. For counterstaining, slides were rinsed and then incubated with 30% hematoxylin for 30 s after developing staining with 3,3′ diaminobenzimidine. All slides were ethanol-dehydrated, cleared with xylene, and mounted with Vectashield mounting medium.

Expression of cardiac and neural markers in embryonic sections was examined by immunofluorescence. E8.5 embryo sections were rehydrated, then subjected to heat antigen retrieval as described above. Sections were incubated with Sox2 antibody (1:200; R&D research, AF2018) from goat and cTNT antibody (1:50; DSHB, RV-C2) from mouse overnight at 4 °C. The following secondary antibodies were used: Alexa 488 anti-goat (1:200; Life Technologies, A-11055) and Alexa 564 anti-mouse (1:500; Life Technologies, A-11037). Slides were mounted with Vectashield mounting media. Images were taken with a Nikon Eclipse 80i microscope and NIS-Elements 4.00.03 software.

### Proliferation and Apoptosis Quantification

For quantification of H3S10P or cleaved caspase 3 immunostaining, digital images were taken using a Nikon Eclipse 80i microscope and the NIS-Elements 4.00.03 software. Positively stained cells were counted manually using ImageJ (v 1.6.0_65). The percentage of H3S10P positive or cleaved caspase-3 positive cells relative to the total number of nuclei was calculated for a minimum of three embryos per genotype.

### RNA sequencing

RNA was isolated from E8.5 embryos using a Direct-zol RNA MicroPrep kit (Zymo research). Enrichment of mRNA, library preparation, and sequencing were performed at BGI, using the BGI-seq format. Reads were mapped to the mm10 genome and quantified using RSEM^27^. Identification of differentially expressed genes was performed using a combined RSEM-EBseq pipeline^20^. Gene ontology enrichment was performed using Metascape (http://metascape.org)^28^.

## Electronic supplementary material

Supplementary Information

Table S1

## Data Availability

Sequence reads for all RNA-seq libraries have been deposited at Gene Expression Omnibus (GEO) and are available with accession number: GSE111691.
