# Supplementary material for: TIP55, a splice isoform of the KAT5 acetyltransferase, is essential for developmental gene regulation and organogenesis
Source: Sci Rep. 2018 Oct 8;8:14908. doi: 10.1038/s41598-018-33213-4 (PMC6175934; doi:10.1038/s41598-018-33213-4)

Supplementary Information for:

TIP55, a splice isoform of the KAT5 acetyltransferase, is essential for developmental gene regulation and organogenesis

Diwash Acharya, Bernadette Nera, Zachary J. Milstone, Lauren Bourke, Yeonsoo Yoon, Jaime A. Rivera-Pérez, Chinmay M. Trivedi, Thomas G. Fazzio

Contents:

Tables S1-S2

Figures S1-S3

Supplementary Tables
Table S1. Changes in gene expression in *Tip55*$^{\Delta/\Delta}$ embryos.

(excel file, provided separately)

Table S2. Primer sequences

RT-PCR primers

Kat5 isoforms (all)	Forward (a): ACGCCACTTGACCAAATGTGA
	Reverse (b): TACTGGCCCTTGTAGTAATTG
Tip55 specific isoform	Forward (a): ACGCCACTTGACCAAATGTGA
	Reverse (c): TACTCACTGAACTCAATAAGC
Tip60β specific isoform	Forward: GATGAGTGGCCCCTGGCTGAGAT
	Reverse: TCCACCTTCCGTTTCACCTCTCT
LTip60 specific isoform	Forward: TGTCTCCCCAGGGGGAGATAATCGA
	Reverse: GGAGGCCGGCACCTCTCTTTC
Gata1	Forward: AGGGCAGAATCCACAAACTG
	Reverse: CCACTAAGGTGGCTGAATCC
Klf1	Forward: CGGGAAGAGCTACACCAAGA
	Reverse: GAGCGAACCTCCAGTCACA
Cited4	Forward: CAGCGCAGAAGAGAGGAGAT
	Reverse: AGTCCCTGCAACTCAACCAG
Mixl1	Forward: CCATGTACCCAGACATCCACT
	Reverse: ACTCTGGCGCCTGGACTT
Hesx1	Forward: CCCAGAACCAGGTCGAAGTA
	Reverse: CCAAATCTGGATTCTGTCTTCC
Gapdh	Forward: TTGATGGCAACAATCTCCAC
	Reverse: CGTCCCGTAGACAAAATGGT

Genotyping primers:

Tip55 tail/embryo PCR	Forward: CAAGTGTTTCCTGGACCACA
	Reverse: TTGCCATAGCCCCGGCGCT

Supplementary Figure Legends

Figure S1. Expression of *Kat5* isoforms in *Tip55*$^{\Delta/\Delta}$ mice.

(A) Schematic diagram of RT-PCR primer binding sites relative to exons present in *Tip55* or all other *Kat5* isoforms. **(B)** RT-PCR with indicated primer sets that detect *Tip55*, all other *Kat5* isoforms, or *Gapdh* as a control. The asterisk indicates a non-specific band in *Tip55*$^{\Delta/\Delta}$. **(C)** Uncropped images of gels shown in (B).

Figure S2. Developmental expression of *Kat5* isoforms. RNA was isolated from wild type E7.5-E11.5 and converted to cDNA using Superscript III reverse transcriptase (Invitrogen). Primers specific for indicated splice isoforms were used for RT-qPCR, and calibrated against known quantities of standards specific for each isoform. Shown are the number of copies of each transcript per 400ng of RNA isolated from embryos at the indicated stages.

Figure S3. Markers of cardiac and neural differentiation are expressed in *Tip55*$^{\Delta/\Delta}$ mice. Immunofluorescence of cardiac troponin T (cTNT) (red) and Sox2 (green) on E8.5 embryo sections of *Tip55*$^{+/+}$ and *Tip55*$^{\Delta/\Delta}$ embryos, taken at 10X magnification to view multiple regions and 20X to focus on either the neural tube (Sox2) or the heart (cTNT).

A

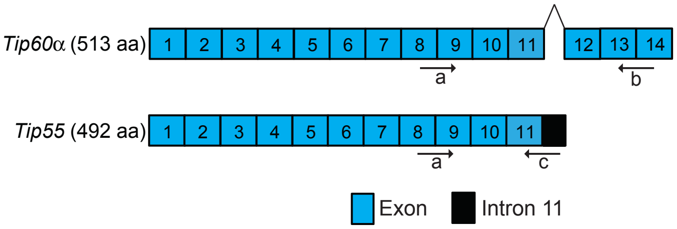

B

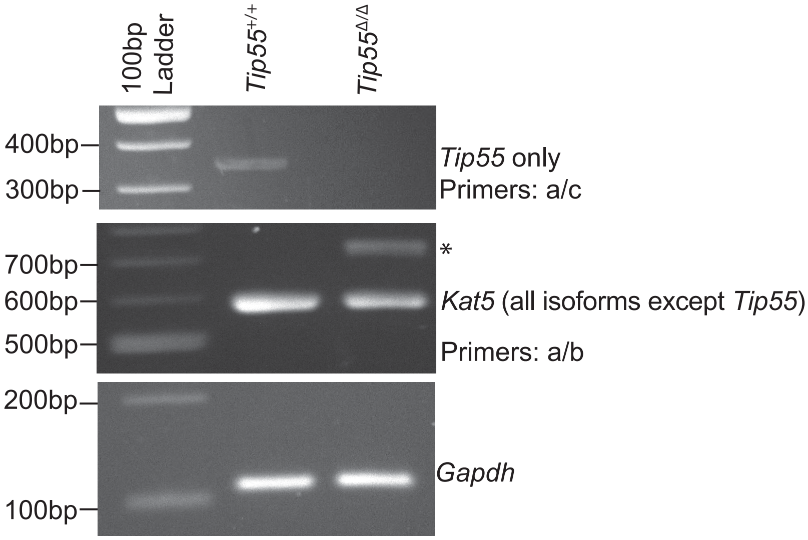

C

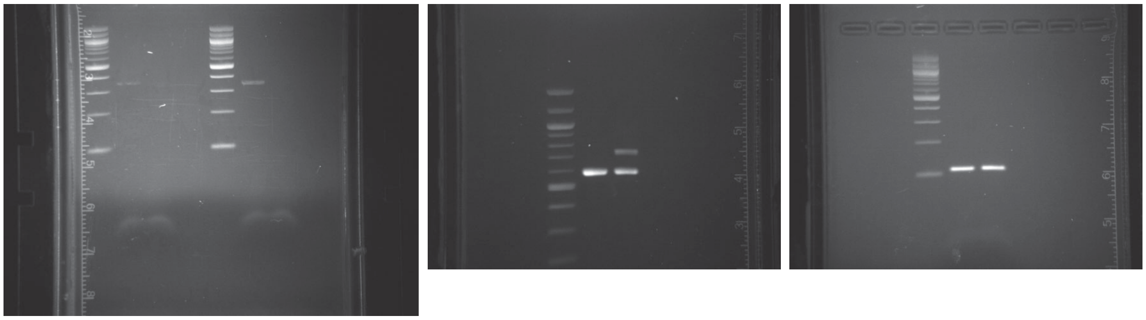

Primers: a/c (Uncropped gel)

Primers: a/b (Uncropped gel)

Primers: *Gapdh* (Uncropped gel)

Figure S2

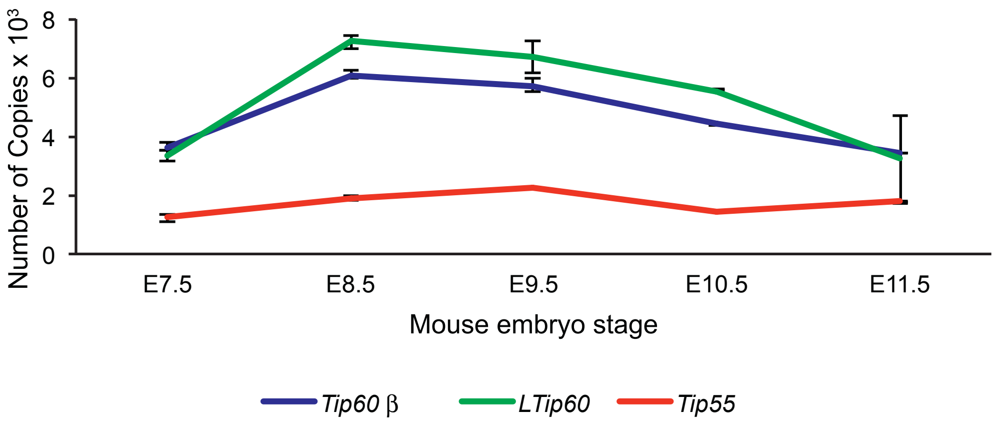
Number of Copies x 10³
8
6
4
2
0
E7.5
E8.5
E9.5
E10.5
E11.5
Mouse embryo stage
Tip60 β
LTip60
Tip55

Figure S3

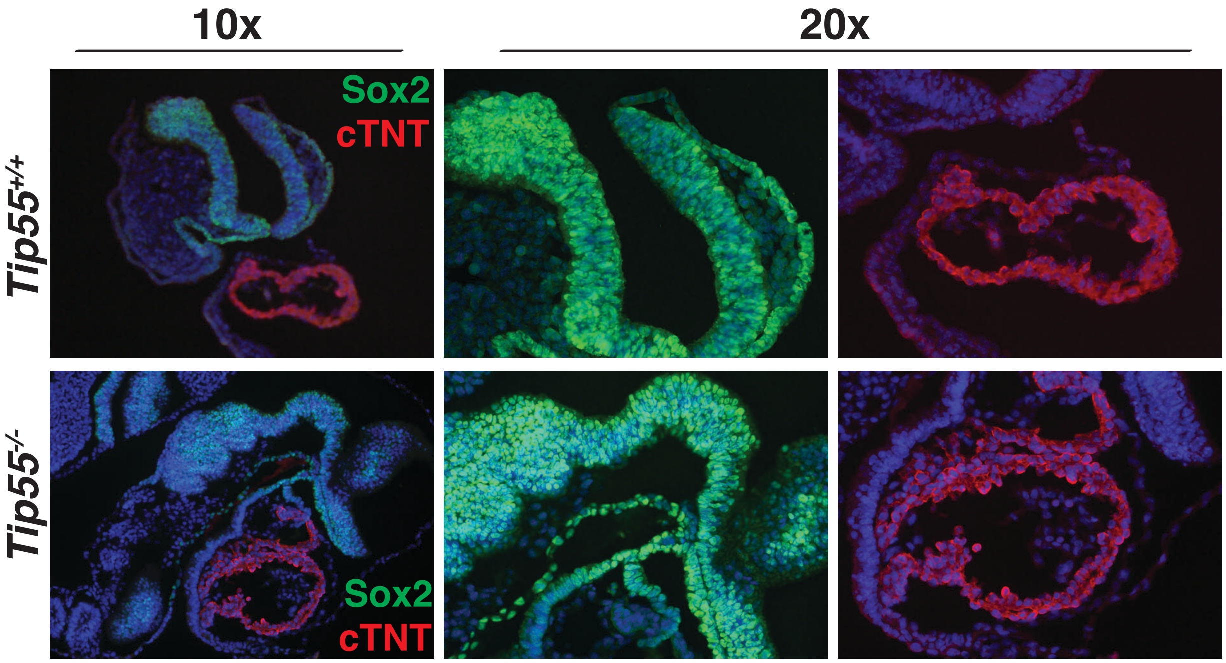

Supplement: Supplementary file 1 — Supplementary Information [file 41598_2018_33213_MOESM1_ESM.pdf]
